# The introduction of mindfulness groups to a psychiatric rehabilitation in-patient setting: a feasibility study

**DOI:** 10.1186/s12888-020-02725-7

**Published:** 2020-06-20

**Authors:** Audrey Millar, Liesbeth Tip, Ruth Lennon, Marlene Macinnes, Beata Michalska, Stephen M Lawrie, Matthias Schwannauer

**Affiliations:** 1 Rehabilitation service, Royal Edinburgh Hospital, Edinburgh, UK; 2 University of Edinburgh, Edinburgh, UK

**Keywords:** Mindfulness groups, Psychosis, In-patient rehabilitation

## Abstract

**Background:**

Patients within psychiatric rehabilitation services have multiple, complex and enduring difficulties, and are frequently described as ‘treatment resistant’. This group have diagnoses of major mental health conditions, most commonly schizophrenia, often alongside a history of complex trauma, co-morbid alcohol/ substance misuse, and cognitive impairment. There is no known effective medical treatment other than Clozapine in this patient group, however, there is preliminary evidence that mindfulness can help individuals with psychosis by improving their ability to cope with stressful internal experiences. This study aimed to determine if mindfulness practice groups are an acceptable therapeutic intervention in an in-patient rehabilitation setting. The study also aimed to monitor the well-being of those who participated.

**Methods:**

Mindfulness practice groups were offered three times weekly on a 15-bedded rehabilitation ward in a psychiatric hospital over 5 months, and weekly in a second ward over an 18 month period. The sessions were delivered by Clinical Psychologists in accordance with adaptations for a psychosis population. Attendance data were gathered on both wards and additional measures of well-being were collected on one ward. Qualitative interviews were conducted with a sample of patients, group facilitators, and staff, to provide supplementary information about the acceptability of the intervention.

**Results:**

In both wards around two thirds (65, 67%) of in-patients attended at least one group and smaller proportion (around a third) went on to attend regularly. There was no discernible impact on well-being using the Warwick-Edinburgh well-being scale. Qualitative interviews suggested a number of benefits to individuals attending as well as the potential for groups to enhance the therapeutic culture within wards.

**Conclusions:**

Clinical guidelines suggest that all patients with a diagnosis of psychosis should have access to psychological therapies, but delivering psychological therapy within an in-patient rehabilitation setting can be challenging. This preliminary feasibility study suggests that mindfulness practice groups are an acceptable intervention, and that further research to look at the effectiveness of mindfulness for symptoms of treatment-resistant psychosis is both possible and merited.

## Background

The population of patients served by psychiatric rehabilitation services are among the most disadvantaged and impaired of any group within the modern NHS. As well as diagnoses of major mental health conditions (most frequently schizophrenia or other psychotic disorders), with ongoing/ residual symptoms; many have a history of complex trauma, co-morbid substance or alcohol misuse, and cognitive impairment. These individuals are often disadvantaged by the difficulties they encounter engaging with mainstream services.

Those who have not benefitted from trials of two or more antipsychotic drugs are designated as having ‘treatment resistant’ schizophrenia (TRS). Within this group some patients exhibit some response to Clozapine, but many are troubled by significant residual symptoms, continue to have frequent, lengthy hospital admissions, and ultimately require support to live independently. A previous study in our service highlighted the complexity of needs within the group requiring in-patient rehabilitation services [1]. Factors associated with the need for prolonged in-patient care (in excess of 6 years) included higher rates of aggression and violence, higher rates of self-harm and suicide attempts, and higher rates of antipsychotic poly-pharmacy, suggesting the presence of ‘treatment resistant’ symptoms. There is a significant economic cost involved in the ongoing care of patients with TRS, as well as an enormous personal cost to patients and their families. At present there is no known effective medical treatment other than Clozapine for TRS.

In terms of psychological treatment, clinical guidelines recommend individual CBTp (cognitive behaviour therapy for psychosis) for the treatment of schizophrenia and schizo-affective disorders [2], whilst the largest evaluation of a group intervention for psychosis has been of Behavioural Family Therapy (BFT) [3]. The bulk of evidence for psychological approaches for psychotic disorders to date has tended to focus on community based patients, and frequently on early intervention. It is only recently that psychological approaches have begun to be adapted for the patient group in in-patient rehabilitation; and the evaluation of such approaches is in its infancy.

Mindfulness meditation focuses on increasing an individual’s awareness of experiences and thinking, helping them to acknowledge habitual reactive responses. An early meta-analysis of the effect of mindfulness-based interventions on psychological functioning across a range of mental health conditions concluded that mindfulness based interventions may help to improve psychological functioning, with a medium post treatment effect size [4].

The number of studies looking at the effect of mindfulness on psychosis specifically has been increasing gradually over the last 10 years, allowing meta-analyses to be conducted. A preliminary meta-analysis evaluated 13 studies which included a mindfulness protocol in the treatment of psychosis [5]. This study found that the interventions were moderately effective in pre-post analysis, in reducing negative and affective symptoms, and in increasing functioning and quality of life (Hedge’s g = .52). When compared with a control group the effect size was smaller (*n* = 7; Hedge’s g = .41).

A subsequent meta-analysis looked at ‘third wave’ interventions for psychosis, where the focus is on one’s responses to difficult experiences and symptoms rather than trying to alter symptoms [6]. The interventions promoted acceptance of difficult experiences, and all incorporated mindfulness as a component. This study examined RCTs only (10 studies), and included two large scale RCTs which were conducted after the previous meta-analysis. These analyses found a moderate between group effect post intervention on depressive symptoms; but no significant effect on positive or negative symptoms, hallucination related distress or functioning. The study concluded that the addition of ‘third wave’ therapies to routine care can improve outcomes in terms of psychotic symptoms, and may be indicated for the treatment of depression in the context of psychosis. Like the previous meta-analysis, the authors noted that effect sizes were smaller when compared with active controls, in particular befriending [7, 8]. They also noted that when studies were grouped by treatment protocol the observed effect on psychotic symptoms appeared greater for protocols including an element of mindfulness based practice than for some of the more complex treatment protocols (for example, Acceptance and Commitment Therapy). Finally, the analyses highlighted between group post intervention differences in measures of mindfulness, leading the authors to postulate that improved mindfulness is the potential mechanism of symptom change.

More recently, preliminary evidence for the effectiveness of mindfulness for psychosis delivered in a group format has emerged. A recent study compared a group intervention which incorporated mindfulness practice and feedback/ discussion with treatment as usual, and found a reduction in voice-related distress, as well as an improvement in depression in the group who received the intervention [9]. This study also found a post intervention reduction in ‘feeling controlled by voices’ in the treatment group.

Overall to date, mindfulness based interventions for psychosis show promise, but the group with ‘treatment resistant’ psychosis remains under-represented in research. An early pilot study in an in-patient setting for the treatment of complex, enduring psychosis trialled a group format incorporating brief mindfulness breathing meditations, and group discussion/ feedback [10]. The group ran for 6 weeks, and eight of eighteen possible participants attended at least once. The study demonstrated that this group of in-patients were able to tolerate short mindful meditations, and to reflect on these experiences.

Despite the early pilot study, and growing evidence that mindfulness-based approaches may be useful in the treatment of psychosis, there has, to our knowledge, been no further research on mindfulness groups for psychosis in an in-patient setting. The in-patient rehabilitation group represents a particular challenge in terms of engagement and the ability to participate in complex psychological treatments. However, the simplicity of mindfulness-based techniques may make these approaches more acceptable. In addition the introduction of mindfulness groups to an in-patient rehabilitation setting has the potential to enhance the therapeutic milieu and improve the patient experience, whilst being a relatively low cost intervention. Building incrementally on the previous pilot study [10], the present study aims to test the feasibility and acceptability of mindfulness practice groups in an in-patient rehabilitation setting, with larger patient numbers, and over a longer time period.

## Methods

### Aims

This study has the following aims;

- To determine if mindfulness practice delivered in a group format is an acceptable therapeutic intervention for in-patients and staff in a Psychiatric Rehabilitation in-patient setting
- To record attendance at mindfulness groups on two wards
- To monitor well-being in those attending groups
- To gather qualitative data on the views of patients and staff who participate in mindfulness groups

### Design

The design was structured evaluation of groups introduced as part of routine clinical practice in two in-patient rehabilitation wards.

### Setting

The study took place within two 15 bedded rehabilitation wards within a major psychiatric hospital. These wards form part of the Psychiatric Rehabilitation service in NHS Lothian. The Psychiatric Rehabilitation service works with individuals who experience multiple disadvantages in all aspects of their lives. The individuals have treatment resistant illnesses (most commonly psychosis), have frequently had adverse early life experiences and have been unable to engage successfully with mainstream health and social services. The service works across in-patient and community settings, and aims to maximise functioning and facilitate community living.

### Participants

Subjects were in-patients within the psychiatric rehabilitation service. Groups were offered to all patients within the two wards studied over 18 months.

The total sample consisted of 6 females and 29 males. The mean age of the sample was 46.3(SD 12.65), and ages ranged from 24 to 67. The length of the current admission in the group ranged from 3 months-120 months (median 35.5 months; mean 44 months). The number of previous admissions of the total group ranged from 4 to 38 (mean 10.9; median 7). The great majority of patients (89%) had a primary diagnosis of schizophrenia (this includes those with schizophrenia and paranoid schizophrenia). The next most common primary diagnoses was schizo-affective disorder (2 patients), and bipolar disorder (2 patients). Secondary diagnoses included poly-substance misuse/ abuse, depression and cognitive impairment (see supplementary Table S1 for more detail on the patient sample).

### Procedure

Mindfulness practice groups were offered three times weekly on one ward over a 5 month period (Ward A). The second ward involved (Ward B) ran weekly groups for a total of 18 months.

Groups incorporated a mindfulness practice led by a Clinical Psychologist, followed by an opportunity for reflection and discussion. The Clinical Psychologists who facilitated the groups were part of the Psychiatric Rehabilitation Psychology service. In addition to doctoral training, facilitators had completed the 8 week mindfulness based stress reduction course, and some had additional mindfulness training as part of another therapeutic model (for example Dialectical Behaviour Therapy, Acceptance and Commitment Therapy). Supervision in mindfulness was incorporated as part of routine clinical supervision.

Groups were delivered in accordance with suggested adaptations for a population with psychosis [11]. Specifically, mindfulness practice was kept brief (10–15 min or less), facilitators used frequent guidance, and routinely made reference to psychotic phenomena such as the experience of voices in the feedback and discussion following the practice. Groups typically lasted for 20–30 min in total.

Groups were open to patients and staff. Part of the reason for this was to encourage attendance, as well as the desire to help the patient group recognise that everyone can use strategies such as mindfulness to manage their mental health.

Qualitative information was gathered using semi-structured interviews with 4 group leaders, 4 staff members who had participated in groups, and 2 patient participants.

As the intervention involved enhancement of routine clinical practice, approval was sought through the Health Board Quality Improvement programme, and all patients who participated gave informed consent.

### Measures

As this was an acceptability/ feasibility study, the main outcome measure was attendance at groups.

On Ward A where there were more frequent groups (three times weekly) for a shorter time period, participants were asked to complete a measure of well-being at the end of each session – the Warwick-Edinburgh well-being scale [12]. In the context of this study, the main purpose was to test the feasibility of measurement in this group, and the experience of clinicians in the service was that patients can be very reluctant to complete questionnaires. For reasons of clinical resource it was not possible to collect this data on Ward B.

Following the pilot period, additional data were gathered using qualitative interviews conducted by the second author, LT, on staff and patient views of the experience of participating in mindfulness groups. An information sheet, and three versions of a semi-structured interview were developed. The purpose of the interviews was to understand more about the experience of participating in the groups, as well as to elicit information about possible benefits and adverse effects of participating, and recommendations for future groups.

## Results

### Attendance data

**Fig. 1:**
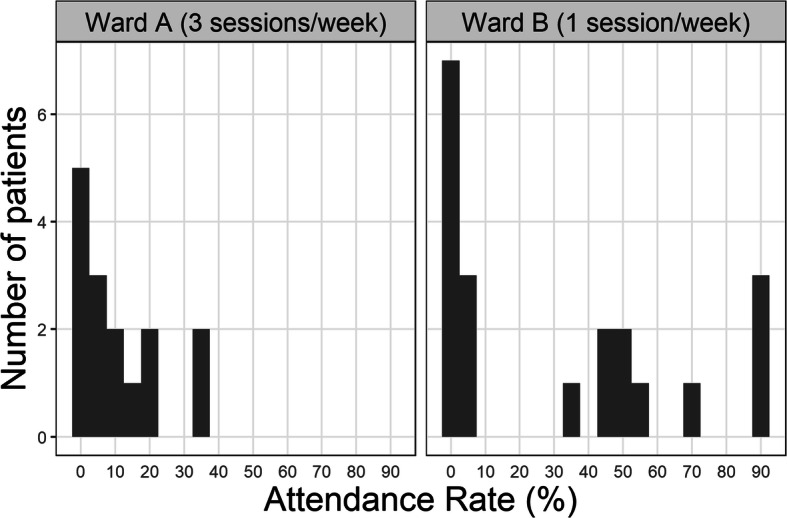
Frequency histogram of attendance rate (number of sessions attended as percentage of possible sessions) for the 15 patients in Ward **a** (left panel) and the 20 patients in Ward **b** (right panel)

The histograms in Fig. 1 show the patient attendance rates for each ward.

#### Ward a

Thirty-three group sessions were run in one ward over a 5 month period, at a rate of 3 sessions per week, although logistical constraints meant that this schedule could not be followed precisely. Every session offered had patients present and the number of patients in each session ranged from 1 to 4 (modal number of patients attending = 2). Ten patients attended group sessions (67% of patients in the ward). Of those who attended sessions, the mean number of sessions attended was 5.2, median number 5 (range 1–12).

On ward A staff also attended mindfulness sessions on 13 of a possible 33 occasions (39% of sessions), and the number of staff who attended ranged from 1 to 3 (modal number of staff attending =2).

#### Ward B

Fifty-seven sessions were run in total over an 18 month period, at the rate of approximately one session per week. All sessions offered had patients present and the number of patients in each session ranged from 1 to 6 (modal number of patients attending = 4). Thirteen out of a possible 20 patients attended at least once (65% of patients on ward). Patients on ward B were offered variable numbers of sessions due to the longer term over which groups were offered and since admissions and discharges occurred within this period, rates of attendance are reported, rather than numbers of sessions attended. The mean rate of attendance was 31.9% (SD =34.6). The median rate of attendance was 19.8% (range 3.5–91.2%). Three participants attended over 90% of available sessions.

On ward B staff also attended on 40 out of 57 possible occasions (70% of sessions). The number of staff per session ranged from 1 to 6 (modal number of staff attending = 2).

Both wards had a small proportion of patients who never attended groups (roughly a third), a larger (roughly two thirds) percentage who attended on at least one occasion and a few patients who went on to attend more regularly. Fig. 1 demonstrates higher rates of attendance in ward B, where groups were offered once a week, than in ward A where groups were held more frequently over a shorter time period. This was also true of staff attendance in Ward B (ie more staff attended more frequently). Detailed information about individual patients’ rates of attendance is available in supplementary Table S1.

**Fig. 2:**
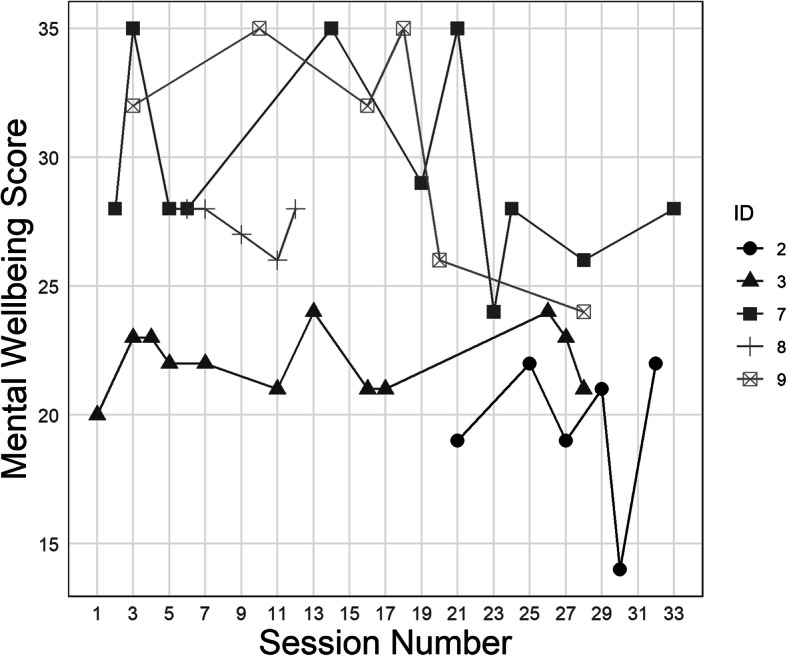
Mental Wellbeing Scale score at each measurement point for the five patients on Ward A for whom five or more measurements were available

Five participants on Ward A completed the well-being scale on 5 or more occasions and these results are illustrated in Fig. 2.

Completed measures showed a considerable amount of variation in well-being over time, but no discernible impact of the mindfulness sessions.

#### Qualitative data

Interviews were conducted with 2 patients who had participated in groups, all of the group facilitators (*n* = 4), and 4 members of nursing staff who had regularly attended groups.

The main themes generated in the interviews, elaborated on below, are as follows:

Adverse effects; engagement; effects and benefits of participating in mindfulness groups (including group processes); challenges/ difficulties.

### Adverse effects

Most interviewees reported no side effects, although one staff nurse commented that patients were more tired following groups.

One theme which emerged was the difficulty of sitting with unpleasant physical sensations, discomfort or emotions (which could include being aware of tiredness):-.

*‘as they’re learning to centre, or ...just focusing on breath, that can bring up a lot of things, and so people can become emotional, or an unsettling thing can maybe happen until they start to ...get used to the whole process of...and know what it’s going to be, or that emotions might come up or something and that that’s okay. So I don’t think that’s necessarily an adversity, but some people might find it uncomfortable ...’ (group facilitator quote).*

This difficulty in ‘sitting with’ things was highlighted in the discussion of challenges of being in the groups, but also mentioned as a potential benefit of practising mindfulness over time by staff and group facilitators.

### Engagement/ things which promote engagement

It is of note that the two patients who consented to be interviewed had continued engaging with mindfulness groups.

The groups were open to all staff and patients on wards and information was provided in several ways on wards. Group facilitators spoke about being on the ward early to try and engage patients. Other factors referred to by interviewees that encouraged engagement in groups were ‘safety’ of the space (and things which helped with this were groups held regularly at the same time and in the same place, and the group facilitator being ‘warm and encouraging’ towards patients regarding attendance). The importance of keeping the mindfulness practice brief (so 10–15 min) was also noted by facilitators, staff and patients.

Facilitators felt that the group could perhaps be appreciated on different levels:-.

*‘I think some of the guys may still struggle with what mindfulness is about and I think some of them, particularly those who might have some more profound cognitive difficulties may just associate the group with it being a kind of relaxation session, but again they still keep coming’ (quote from group facilitator).*

Feelings about the level of self-disclosure involved/ required in the group were somewhat conflicted; for example one facilitator felt that compared to other groups and activities the mindfulness group required fairly minimal contributions from patients:-.

*‘(patients) didn’t really interact because it was a meditation, you know, they were just focusing on themselves and their own breath. So, you know it’s very different to a psychodynamic group, or a CBT group where people are interacting and sharing with each other, whereas people were just coming in, and sitting down, and engaging with meditation and then, sort of, leaving..’ (quote from group facilitator).*

This was supported by a comment from one of the nurses:

*‘I think the mindfulness groups compared to maybe some of the more structured therapy groups...I think mindfulness is more accessible for some people, it’s short, I think it’s less threatening, I think people are aware they can come and sit and they can go through the practice and they don’t have to give feedback if they don’t feel comfortable, they can just listen to other people’s feedback.’ (quote from staff nurse).*

On the other hand, one of the nurses interviewed commented that one of the positive things about the group was that most people contributed, and gave feedback at the end of the practice:-.

*‘I think it’s great how she (group facilitator) has time for feedback at the end. I think it’s good if they have anything on their chest and they want to get it off, they’re allowed to at the end so, yes, and they feel comfortable doing it...’ (quote from staff nurse).*

It may be that the groups could function on two levels; and for some participants just sitting in the group and participating in the exercise could be relaxing, whereas for other group members the full experience involved sharing with other group members.

It was mentioned that groups would ‘evolve’ and then have a core group of the same patients attending over time, which added to the feeling of a ‘safe’ group:-.

*‘I think once the mindfulness group was established, even though we had people who were discharged, because we always had a couple of core members, that once the group was established, it’s much easier to get people to come along,..’ (quote from group facilitator).*

This fits with the data on frequency of attendance, and the fact that on each ward there appeared to be a fairly large group of patients who were prepared to try the group, and a smaller sub-group who attended repeatedly. It was also mentioned by group facilitators and staff that the ‘accepting’, tolerant, encouraging and flexible attitudes of group leaders towards attendance promoted engagement:-.

*‘I think (the group leader) is really kind. I think she’s really warm with all the patients. I think they all feel included and I don’t think anyone would feel like...it doesn’t feel like they’re forced to come in which I think is good for them because I know that there have been a couple of patients who maybe have only stayed for part of a session and she never makes them feel like they can’t come back in ...’ (quote from staff nurse).*

This inclusion of group members even where they had missed groups, or had to leave, seemed to create a feeling of tolerance and acceptance of difficult experiences as part of life and part of the culture of the groups; all of which could be hypothesised to promote engagement.

Staff, group facilitators and patients who were interviewed all thought that having the nursing staff also invited to attend was beneficial in promoting engagement for patients, as patients often had trusting relationships with staff and felt encouraged by staff being present:-.

*‘I think as well when nursing staff come along, that it can be helpful for patients to feel safer...when they first started coming they didn’t know me very well and I think having nursing staff there made it easier for a lot of the guys to come in and feel safe in the space..’ (quote from group facilitator).*

*‘I think for certain patients it gives them a bit of comfort if there’s a member of staff they’re familiar with so I think that part of it’s good, it makes them feel more relaxed.’ (quote from staff nurse).*

*‘I suppose staff joining in helps a lot...’ (quote from patient).*

Having nurses there seems to have lowered the threshold for patients attending. The staff who were interviewed had a range of experience and levels of knowledge of patients – (staff had known patients from between 10 weeks and 7 years).

*‘..I think they’re more likely to try something out if they’ve got a very strong relationship with the nursing staff member who’s suggesting this...I think because the relationships with nurses are their primary relationships’ (quote from group facilitator).*

The inclusion of staff also meant that staff could explain a bit about groups to patients who might participate in future, and that staff were aware of what happened in the groups and of the experience of being in the group. This could help to reinforce aspects of the group, but also could contribute to bonding between patients and staff (thus further building trusting relationships, and enhancing the therapeutic culture on wards).

Two members of staff commented on feeling they understood patients better from attending the groups. It was also observed by group facilitators that participating in mindfulness groups could encourage patients to engage further with psychology staff, and consider participating in further psychological therapy:-.

*‘I think it’s a nice way to introduce people to the idea of what therapy might be like without actually having to be fully committed to it. I think the mindfulness works, the way we run the mindfulness group on (ward) works really well as a stepping stone into familiarising people into what therapy might be like.’ (quote from group facilitator).*

### Effects/ benefits of engaging in mindfulness groups

The potential to enhance the therapeutic culture on wards was one benefit that was frequently alluded to in the interviews, both in terms of building or enhancing bonds between patients, between staff and patients; and also in terms of attendance at mindfulness groups making it more likely that someone would go on to engage in one to one therapy:-.

*‘I think one of the very important things for patients is to build relationships and find a little bit of comfort through going through similar difficulties with other people. From this perspective the group feels two purposes because it enables us to provide structure to the day....On the other hand it also enables people to take part in something together...”(quote from staff nurse).*

*‘I think when you share a group, when you meet, especially seeing the same people more than one time, over time, that you do have a shared bond, a similarity, a trust building that goes on...that’s similar to other groups. But mindfulness probably, I don’t know,...it’s hard to explain...you’re making yourself vulnerable in a way that’s slightly more deep, personal..’(quote from staff nurse).*

*“...I think there may be a bidirectional relationship between participating in one to one therapy and participating in the group therapy....and I think what seemed interesting was that some patients who initially refused to be seen for one to one therapy were encouraged by other patients to attend the group. Having had the experience of participating in the group and getting to know the therapist, they were more willing to then engage in one to one therapy and vice versa....Obviously there could be a sense of validation through being in the group and seeing that there are other people who value it and who find it meaningful.” (quote from group facilitator).*

*“..what you share afterwards, all of that, that ability to be open with that person, and then they’re sharing it with you. And I think that is very therapeutic probably for them; it is for me, I mean I really feel that we have that unique connection as well, so it’s comforting too.” (quote from staff nurse).*

Several other benefits of participating in mindfulness groups were described by staff and patients. Staff and patients referred to being ‘*more relaxed’*, less distressed, and ‘*calmer’ ‘more grounded’* after sessions. People also referred to having a ‘*clearer mind*’ after the sessions:-.

*‘(After a group) some of the patients will feel tired...and they’ll be less seeking extra medication’ (quote from staff nurse).*

*‘I suppose it clears their mind for that space of time and I suppose relaxes them in a way’ (quote from staff nurse).*

*‘...I certainly observed participants coming to the sessions distressed and ending the session feeling much less distressed. So I could see an immediate effect of the stress levels going down for some of the participants’ (quote from facilitator).*

One of the patients who regularly participated described benefits and in particular the cumulative benefit of participating.

*‘At first I was very distracted, by the very painful feelings....last time I was quite surprised because there was much less distraction...’ (quote from patient).*

One patient spoke of learning to focus on breathing and other aspects of bodily experience instead of painful experiences.

*‘But I’m in a lot of pain and it’s the first time, last Wednesday...a few days ago that I could focus on my breath at all’ (quote from patient).*

Nurses also referred to the cumulative benefits:-.

*‘I really enjoyed it, as a person, just on my own...Every time, I think that you ...I think that...all my past experience of doing it is still sort of there, which I found fascinating for me, that I can sit down in a group somewhere and having done it before, that past practice allows me to go into it’ (quote from staff nurse).*

Staff interviewed were very positive about the benefits of participating for themselves as well as for the effects on their relationships with patients:-.

*‘You’re kind of sleepy, drowsy like while it’s going on but afterwards you’re kind of refreshed I suppose’ (quote from staff nurse).*

*‘I loved it...It helped me sort of re-engage all the time...engage with my body again...oh I’ve been breathing too fast or I’ve been worrying about such and such...’ (quote from staff nurse).*

Several of the benefits of engaging with the groups seemed to relate to the group processes. In particular, ‘acceptance’ and normalisation were mentioned.

The idea of acceptance and tolerance of difficult experiences as the crucial part of what is being practiced came through strongly in the interviews.

*‘...so generally quite a lot of the guys will be able to talk about the fact that they were aware of what was going on internally for them...so...they were aware of the voices they were hearing or the difficult thoughts or emotions that were going on and being able to be aware but without being actively involved in it,...being able to notice what was going on without getting drawn into it..’ (quote from group facilitator).*

*‘..the long term is about getting to know when you are distressed and when you’re not distressed. So I guess it’s about monitoring your own....to just sit and ...be friends with your thoughts, rather than trying to monitor them...well you are monitoring but not trying to modify them as if they were doing the more CBT approach’ (quote from group facilitator).*

Two group facilitators commented that being mindful may help patients to feel more ‘*in control*’ of their psychotic experiences.

Normalisation is a recognised benefit of all groups, however this may carry particular value in an in-patient setting in reducing stigma and isolation.

*‘I think there are a couple of people who’ve come to mindfulness groups where, I think that they haven’t necessarily had the option or the experience of talking to other people about psychosis...coming to the group might have been the first time that they heard other people mentioning that they heard voices and I think that was really powerful for them.’ (quote from group facilitator).*

*‘part of mindfulness is about normalising human experiences and I think that that really helps because the feedback that we do at the end of the session is feedback from everybody, so I’ll encourage nursing staff and other staff who come along to give feedback too and it can just be about acknowledging what was going on for them in that practice, be it their mind was busy or that they were aware they were feeling anxious or breathing too quickly’ (quote from group facilitator).*

*‘well maybe it’s something about...we’re all in the same boat,...we’re sharing this together... it’s part of the human experience to have all this going on...’ (quote from group facilitator).*

*‘I think it’s good for the patients to see a staff member doing it as well...also to get rid of “them and us”...’ (quote from staff nurse).*

A further group process and benefit of having patients and staff attend together which interviewees identified was the process of modelling:-.

*‘...the patients were pleased the members of staff were in the group as well. It seemed to be really good modelling’ (quote from group facilitator).*

As well as staff-patient modelling, patients modelling each other was referred to:-.

*‘I guess it could have worked as well because ...I guess modelling...you see other patients are doing it, maybe if you’re reluctant to do it, you might give it a chance because actually your pal over there is doing it...’ (quote from group facilitator).*

This effect of increased mindfulness over time, and the cumulative benefit of participating was mentioned in interviews with facilitators, staff and one of the patient interviews.

*‘It’s been very helpful...it’s been a cumulative effect..It’s actually got better’ (quote from patient).*

*‘she said at the start she didn’t feel it was benefitting her...as she gradually went to more, she found herself feeling...paying more attention’ (quote from staff nurse describing a colleague’s reactions to the group).*

*‘You have your moments where you feel your mind kind of wandering but I’m always able to come back and focus...before I wasn’t able to do that’ (quote from staff nurse).*

This cumulative effect and benefit connects with the observations highlighted earlier that patients and staff who take part may become better at noticing their experiences (and these could be bodily/ physical experiences, for example connected to breathing or pain, or they could be internal, mental experiences, such as thoughts or the experience of hearing voices).

### Challenges and difficulties

As well as a number of benefits of the sessions, facilitators and staff in particular reported a number of challenges with the mindfulness groups:-.

Difficult dynamics between patients and other factors that reduce safety on the ward, (such as drug and alcohol use by patients), can be brought into the mindfulness sessions.

Group dynamics can occasionally inhibit group members attending, and dynamics, in interaction with psychosis symptoms can hinder patient’s concentration during a group. On the flip side of the difficult dynamics, friendships between particular patients could also affect the group dynamic. There was however a recognition that these factors were part of the life and dynamics of the ward, and shouldn’t prevent groups from happening. Facilitators saw it as their job to ‘hold the boundaries’ of the group and make the group safe.

From the facilitators’ point of view, having predictable times and settings for groups helped with this, as did the presence of nursing staff within groups.

## Discussion

Previous reviews allude to the complexity of psychological therapies as a potential obstacle to engagement for patients with significant psychotic symptoms [6]. The in-patient population in rehabilitation represents the greatest challenge in engagement of all patients with psychosis. Many have lived in hospital for significant periods, with ongoing delusions and other positive symptoms, as well as significant negative symptoms. There is a need to offer therapeutic activities which are acceptable in this setting.

This study found that the majority of in-patients in a Psychiatric Rehabilitation setting (roughly two thirds) are prepared to try mindfulness practice groups, and this compares very favourably with other therapeutic activities on offer on wards from psychologists and other members of the multi-disciplinary team. A smaller group (roughly a third of in-patients) go on to attend regularly, and this study demonstrated that this can be sustained over a prolonged (eighteen month) period. In fact the attendance data from the two wards as highlighted in Fig. 1 seems to indicate that over a prolonged time period there is more opportunity for these groups to become embedded within the culture of the wards, as there was a smaller group of patients who continued to attend very regularly over a much longer time (with 3 patients attending over 90% of sessions in Ward B). Again this compares very favourably with engagement in other activities within an in-patient rehabilitation setting.

The staff group on both wards also engaged and attended the groups, with rates of attendance being better and attendance by staff being sustained in Ward B where the groups happened weekly over a longer (18 month) period. There seemed to be no advantage to offering more frequent groups, as in fact the rates of attendance were better in Ward B. This is an important finding for implementation, since weekly groups are likely to be a more sustainable option in rehabilitation services, where small numbers of staff are available to run therapeutic groups.

Qualitative data on engagement suggest that several factors may promote engagement in the groups, including an encouraging, warm and accepting attitude of the facilitator, the sense of a ‘safe’ space, and the inclusion of nursing staff in the groups. Feedback in qualitative interviews also suggests that keeping mindfulness practices brief (up to 15 min), and having a repertoire of different activities and practices may increase engagement.

Qualitative interview data suggest minimal adverse effects for those attending mindfulness groups. Noticing increased tiredness was one effect mentioned for patients and staff. Additionally, some of the difficulties of ‘sitting with’ difficult experiences were highlighted, whether those be bodily sensations (such as pain or tiredness), or difficult internal experiences such as thoughts and worries. Rather than being an adverse effect, it can be argued, this is central to the philosophy of mindfulness practice [13]. However, as only two patients consented to be interviewed, it is possible that others who declined may have reported adverse effects. On balance, we are reassured that, despite issues with completing questionnaires or agreeing to interviews, many patients continued to attend, indicating acceptability of the intervention whilst underlining the challenges of measurement in this setting.

Several benefits of the mindfulness groups were highlighted in the interviews. Patients, staff and group facilitators all referred to the potential for the groups to make them feel calmer or more grounded. There was also reference to a reduced need for ‘as required’ medication following mindfulness practice groups, and although we are unable to quantify this, it is something worth measuring systematically in future studies. Frequent references were made to the possibility of groups enhancing the ‘therapeutic culture’ in wards, and this was hypothesised to be through the effects of the groups on relationships (both patient – patient relationships and relationships between patients and staff); and also through encouraging patients to go on to take part in further therapy or additional therapeutic activities. This potential for something as simple as a regular mindfulness practice group to ‘activate’ or encourage patients to engage with ‘treatment’ in a wider sense is something which could also be of considerable value in rehabilitation in-patient settings, where patients’ levels of negative symptoms and low motivation are sources of difficulty for patients and staff.

Several other group processes were also highlighted as being of benefit. The concept of ‘acceptance’ featured frequently –in reference to both an accepting attitude towards others in the group (as modelled by the group facilitator), and acceptance or tolerance of difficult experiences such as pain, discomfort or difficult thoughts or internal experiences. This echoes mindfulness theory about the purpose of mindfulness meditation [13], as well as supporting earlier findings [9]. This previous study in an out-patient group demonstrated an increased ability to tolerate psychotic experiences following group mindfulness practice. This also links with theory about the cumulative benefits of mindfulness practice, in terms of an increased ability to accept experiences as well as to detach value from different experiences.

Other group processes highlighted as being of potential value included normalisation and modelling. The inclusion of staff within groups was seen as exerting a normalising effect, in terms of highlighting shared and universal human experiences of difficulty, suffering, and pain. The ability of patient group members to share experiences, for example around voice hearing, was also seen as normalising and of benefit. Modelling was also mentioned in terms of patients witnessing others attending groups, and witnessing other patients, staff and facilitators sharing their personal experiences of mindfulness practice in the feedback afterwards. These are recognised group processes and benefits in many group interventions, however these may be particularly powerful processes in the context of a psychiatric in-patient setting, in overcoming stigma and isolation.

Qualitative data highlighted difficult group dynamics on the ward, and drug and alcohol use by patients as features which could make groups more difficult to facilitate. As well as this the challenge of measurement was highlighted by group facilitators in interviews. It is of note therefore that measures were completed on the vast majority of occasions where patients attended on Ward A over the 5 month period. For reasons of clinical resource in the service we were not able to obtain measures in Ward B, and it is unclear how meaningful measures taken on Ward A were. Unfortunately there are also too few repeated measures of well-being obtained to reach any clear conclusions about the impact of the groups on well-being over time, although Fig. 2 suggests that the groups have not had any discernible negative effect on well-being, and the fact that patients were prepared to continue engaging with groups over a longer time period supports this. It may be that simple visual analogue measures, to be completed before and after sessions, would have been less burdensome than the well-being scale.

This difficulty obtaining meaningful measurement is something which clinicians in the service are aware of, and is perhaps one of the obstacles to research being done in in-patient settings. This general problem was also noted in the previous pilot study [10]. Review and meta-analysis articles highlight the heterogeneity of studies in terms of measures used to quantify change in patients with psychosis. Studies also vary in terms of whether they use measures of symptoms or measures of distress as primary outcomes. Further work is needed to explore and agree on a set of measures to evaluate the clinical effects of mindfulness practice, as well as to identify useful measures for an in-patient setting.

This study only aimed to test feasibility, but has several limitations including a single specific site, relatively small numbers of participants and the challenges outlined with obtaining meaningful measurement. Nonetheless, the study provides a starting point, and suggests that the intervention is feasible and may be of benefit as indicated in qualitative data. A larger scale multi-site trial may be warranted.

## Conclusions

Clinical guidelines suggest that all patients with a diagnosis of psychosis should have access to psychological therapies, but the patient group in psychiatric rehabilitation in-patient settings can present a particular challenge in terms of engagement and participation in therapy. This preliminary feasibility study suggests that brief mindfulness practice groups are an acceptable intervention, that two thirds of patients are prepared to try mindfulness groups and that a smaller proportion go on to attend frequently and report some benefits. Several features of groups, in particular the inclusion of staff as well as patients in groups, may be of benefit in promoting engagement. The study doesn’t suggest any detrimental effect on well-being from participating in these groups.

In conclusion; this study indicates that implementation of mindfulness groups in psychiatric rehabilitation wards is feasible and of potential benefit, and that this approach could be adopted in multiple sites. Further research to examine the specific effects and impact of mindfulness practice on psychosis, and the distress experienced by patients with severe and enduring symptoms of psychosis is merited and worthwhile.

## Supplementary information

**Additional file 1.** Millar_mindfulness_attendance_rate_data

**Additional file 2.** Millar_mindfulness_mental_wellbeing_scale_data

## Data Availability

All quantitative data-sets generated or analysed during this study are included in this published article and its supplementary materials. The qualitative data-sets are available from the corresponding author by reasonable request.

## Abbreviations

TRS: Treatment Resistant Schizophrenia
CBTp: Cognitive Behaviour Therapy for psychosis
BFT: Behavioural Family Therapy
RCT: Randomised Controlled Trial
CBT: Cognitive Behaviour Therapy
